# *Bacillus* spp. Probiotic Strains as a Potential Tool for Limiting the Use of Antibiotics, and Improving the Growth and Health of Pigs and Chickens

**DOI:** 10.3389/fmicb.2022.801827

**Published:** 2022-02-07

**Authors:** Diana Luise, Paolo Bosi, Lena Raff, Laura Amatucci, Sara Virdis, Paolo Trevisi

**Affiliations:** ^1^Department of Agricultural and Food Sciences, University of Bologna, Bologna, Italy; ^2^Chr. Hansen, Animal Health and Nutrition, Hørsholm, Denmark

**Keywords:** antibiotics, *Bacillus*, gut health, pig, probiotics, broiler

## Abstract

The pressure to increasingly optimize the breeding of livestock monogastric animals resulted in antimicrobials often being misused in an attempt to improve growth performance and counteract diseases in these animals, leading to an increase in the problem of antibiotic resistance. To tackle this problem, the use of probiotics, also known as direct in-feed microbials (DFM), seems to be one of the most promising strategies. Among probiotics, the interest in *Bacillus* strains has been intensively increased in recent decades in pigs and poultry. The aim of the present review was to evaluate the effectiveness of *Bacillus* strains as probiotics and as a potential strategy for reducing the misuse of antibiotics in monogastric animals. Thus, the potential modes of action, and the effects on the performance and health of pigs (weaning pigs, lactation and gestation sows) and broilers are discussed. These searches yielded 131 articles (published before January 2021). The present review showed that *Bacillus* strains could favor growth in terms of the average daily gain (ADG) of post-weaning piglets and broilers, and reduce the incidence of post-weaning diarrhea in pigs by 30% and mortality in broilers by 6–8%. The benefits of *Bacillus* strains on these parameters showed results comparable to the benefit obtained by the use of antibiotics. Furthermore, the use of *Bacillus* strains gives promising results in enhancing the local adaptative immune response and in reducing the oxidative stress of broilers. Fewer data were available regarding the effect on sows. Discordant effects have been reported regarding the effect on body weight (BW) and feed intake while a number of studies have supported the hypothesis that feeding probiotics to sows could benefit their reproductive performance, namely the BW and ADG of the litters. Taken all the above-mentioned facts together, this review confirmed the effectiveness of *Bacillus* strains as probiotics in young pigs and broilers, favoring their health and contributing to a reduction in the misuse of direct in-feed antibiotics. The continuous development and research regarding probiotics will support a decrease in the misuse of antibiotics in livestock production in order to endorse a more sustainable rearing system in the near future.

## Introduction

Livestock monogastric species, including swine and poultry, are the main sources of meat; therefore, they are, at the same time, the main livestock species reared under intensive conditions (Eurostat, 2022). The pressure to increasingly optimize breeding resulted in antimicrobials often being used for both prophylactic and metaphylactic purposes to counteract the diseases of these animals or as growth promoters. It is currently commonly recognized that the misuse of antibiotics over a long period of time causes selection pressure on the bacteria which leads to an increase in antibiotic resistance and the loss of environmental microbial diversity (Aidara-Kane et al., 2018; Checcucci et al., 2020). Furthermore, several recent investigations have reported the emergence of multidrug-resistant bacterial pathogens derived from different livestock origins, including chickens, pigs and cattle (Algammal et al., 2020, 2021; Kimera et al., 2021). Therefore, the need for the proper use of antibiotics as well as the application of new safe alternatives is urgent. The European Union has included this issue in the main points of the farm to fork concept and, in the near future, the European Commission will take action to reduce the overall sales of antimicrobials for livestock animals in order to achieve a 50% reduction by 2030 (European Commission, 2020). To tackle this issue, researchers, together with the stakeholders involved in livestock production, have been called upon to identify sustainable solutions to replace and reduce the misuse of antibiotics in livestock. Of the natural and alternative feed additives, the use of probiotics, also called direct in-feed microbials (DFM), seems to be one of the most promising strategies as highlighted by several reviews (Barba-Vidal et al., 2019; Cameron and McAllister, 2019; Neveling and Dicks, 2021). Furthermore, probiotics have been extensively associated with an improvement in human and animal health in many different ways; namely, having a positive effect on gut immunity by regulating the composition and metabolism of gut microbes, improving the digestion and absorption of nutrients and inhibiting the potential pathogenic bacteria, thus regulating intestinal disease (Ding S. et al., 2021; Savitri, 2021).

The ideal characteristics of a specific probiotic for livestock include increasing the productivity and the health of the animals; however, at the same time, it needs to be recognized as not being drug or multidrug resistant bacteria which are able to survive in the gastrointestinal environment (low pH and bile acids) (Bajagai et al., 2016). Additional characteristics also include their capacity to adhere to the intestinal mucosa (thus, to act as a direct competitor of potential pathogens) and to grow rapidly. In addition, probiotics need to tolerate the manufacturing, transport and storage processes which are usually applied to feed and, thereafter, to maintain their vital characteristics (Bajagai et al., 2016). For the latter characteristic, spore forming bacteria are particularly indicated since they are able to maintain their vitality after feed pelleting, storage and manipulation (Cutting, 2011).

Of the different commercially available probiotics, many strains of *Bacillus* spp. are currently being used as probiotics for monogastric animals. Bacillus are gram-positive, catalase-positive, spore-forming, aerobic and facultative anerobic bacteria which distinguishes them from Clostridia and sporolactobacilli. At the moment of writing this review, a total of 2,552 species of *Bacillus* spp. have been recognized.^1^ The *Bacillus* species which have been most extensively examined as probiotics for monogastric animals have been most commonly isolated from soil and from the gastrointestinal tracts of animals, and are *Bacillus subtilis*, *Bacillus licheniformis*, *Bacillus coagulants*, *Bacillus amyloliquefaeciens*, *Bacillus velezensis* and *Bacillus cereus*. These *Bacillus* probiotic species have been proven to possess several properties and abilities (such as the capacity to produce antimicrobial molecules and enzymes), together with the capacity of sporulating, the latter extends their period of effectiveness and gives them a double advantage in terms of survival (heat tolerance and longer shelf-life) in diverse environments as compared to other probiotics, such as Lactobacillus (Abriouel et al., 2011; Mingmongkolchai and Panbangred, 2018).

Therefore, in recent decades, interest in *Bacillus* strains as a probiotic has been intensively increased, and several applications have been studied in pigs and poultry (Mingmongkolchai and Panbangred, 2018; Li et al., 2019, 2021; Ding S. et al., 2021). The aim of the present review was to evaluate the current knowledge regarding the effectiveness of *Bacillus* strains as probiotics and as a potential tool for reducing the prophylactic and metaphylactic use of antibiotics in pigs and chickens; thus, the potential modes of action, and the effects on the performance and health of pigs (weaning pigs and lactating and gestating sows) and poultry (broilers) were considered.

## Mode of Action of *Bacillus* spp. Probiotic Strains

**FIGURE 1:**
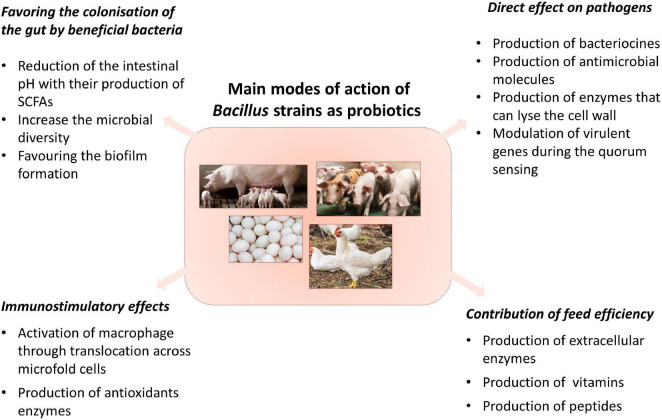
Principal modes of action of Bacillus probiotics.

In monogastric animals, one of the main interests is the relationship between nutrition and gut health, especially in the small intestine. In fact, as recently proposed by Chalvon-Demersay et al. (2021), four main interconnected pillars [namely (1) barrier function and absorption, (2) intestinal immune fitness, (3) oxidative stress homeostasis and (4) microbiota balance] need to be controlled for proper gut health. *Bacillus* strains applied as probiotics have several modes of action which can affect one or more of the aforementioned pillars. The main modes of action of probiotics have been evidenced by *in vivo* and *in vitro* studies, and have been described in some reviews (Chaucheyras-Durand and Durand, 2010; Vilà et al., 2010). The modes of action of Bacillus probiotics are shared and show synergies among strains; Figure 1 aims to depict the complexity of the main modes of action of *Bacillus* strains as probiotics. This section presents a brief description of the main modes of action and some examples targeted at pigs and poultry.

### Direct Effect on Pathogens

Bacillus probiotics can have a direct effect on pathogenic bacteria, resulting in prevention, inhibition or arrest of their growth and colonization of the gut. The direct effect on pathogens could be due to the production of antimicrobial peptides and metabolites, including bacteriocins which have antagonistic effects against pathogenic microorganisms. Bacteriocins are a heterologous group of proteinaceous antimicrobial substances which can exert their effect against specific bacteria, although many of them have shown a wider spectrum of activity (Abriouel et al., 2011). For instance, *B. subtilis* was initially known to produce subtilin, a bacteriocin (Jansen and Hirschmann, 1944) which has a broad antimicrobial effect against several gram-positive bacteria via permeabilization of their cytoplasmic membrane (Kordel et al., 1989). Subsequently, several additional bacteriocins produced by different *B. subtilis* strains were identified, including sublancin 168 (Paik et al., 1998), bacillocin 22 (Zheng and Slavik, 1999) and subtilosin A (Shelburne et al., 2007). *B. licheniformis* can produce a wide range of bacteriocins, depending on the strains which include bacteriocin-like inhibitory substance (BLIS) (Cladera-Olivera et al., 2004), bacillocin 490 (Martirani et al., 2002) and L8 (Smitha and Bhat, 2013) which function mainly against gram-positive bacteria or bacteria very close to the Bacillus family (for bacillocin 490). *B. amyloliquifaciens* can produce subtilosin (Sutyak et al., 2008) and CAMT2 (An et al., 2015). A detailed classification and description of the bacteriocins produced by *Bacillus* spp. Have been exhaustively reported by Abriouel et al. (2011). More recently, thanks to the availability of whole genome sequence data, additional antimicrobial proprieties related to the production of secondary metabolites, including non-ribosomally synthesized peptides and polyketides have been identified for several *Bacillus* strains, namely *B. subtilis*, *B. amyloliquefaciens*, *B. velezensis* and *B licheniformis.* A comprehensive review of these properties has been reported elsewhere (Harwood et al., 2018; Blin et al., 2019). In particular, some non-ribosomally synthesized peptides, including surfactins, fengycin, fusaricidins and iturins produced by *Bacillus* strains, have been demonstrated to have antifungal properties (Harwood et al., 2018; Vasilchenko et al., 2022). Evidence regarding the antifungal proprieties of *Bacillus* strains is mainly based on plants and soil (Ongena et al., 2005; Ongena and Jaques, 2008). Siahmoshteh et al. (2018) reported that *B. subtilis* and *B. amyloliquefaciens* have antifungal actions against aflatoxigenic *Aspergillus parasiticus* which can produce hazardous toxins in several feedstuffs and crops, causing chronic diseases in human and animals. More recently, *B. velezensis* JT3-1, isolated from yak feces, showed antimicrobial and antifungal proprieties (Li et al., 2020).

In addition to the production of bacteriocins and antimicrobial molecules, *Bacillus* spp. probiotics can exert a direct effect on pathogens by modulation of their virulent genes during a process called quorum sensing (QS). During the quorum sensing process, bacteria produce signaling molecules (autoinducers) which induce a response at a high cell density via activation of downstream gene expression (Miller and Bassler, 2001). Among these signaling molecules, N-acyl homoserine lactone (AHL) is known to be an autoinducer, connected to the expression of virulence genes. The *Bacillus* strains were the first bacteria in which the AHL-degrading enzyme was identified (Dong et al., 2002), raising interest in these genera as a regulatory factor in the QS process. In addition to that, Bacillus is also known to produce enzymes which can interfere with bacterial QS molecules, including AiiA and P450BM-3 (Chen et al., 2013).

An additional way for the direct inhibition of pathogens by *Bacillus* spp. is related to their capacity to produce enzymes which can lyse the cell wall of pathogenic bacteria, including elastase and endopeptidases. For instance, *B. subtilis* 6 caused the lysis of freshly grown cells of Gram-negative bacteria, including *Salmonella typhi* and *Klebsiella pneumoniae* by the production of elastase (Gupta et al., 1992). *B. licheniformis* YS1005 had a highly lytic activity against *Streptococcus* mutans strains by the production of two endopeptidases (Kim et al., 1999).

### Favoring the Colonization of the Gut by Beneficial Bacteria

In addition to the direct effect on pathogens, probiotics are known to promote gut eubiosis, favoring colonization of the gut by means of commensal and non-pathogenic bacteria by modulating the gut environment. For instance, *Bacillus* spp. probiotics can reduce intestinal pH by means of the production of short chain fatty acids (SCFAs), including lactate (Wang et al., 2011; Ciurescu et al., 2020). A proper balance of intestinal microorganisms is crucial for the maintenance of the animal’s health and performance at every stage of the animal’s growing phase (Trevisi et al., 2021). Currently, for the most part, the alpha (Chao, Shannon, Simpson indices) and beta diversity indices are used to describe the status of intestinal eubiosis. Generally, an increase in these indices has been associated with a more resilient microbiota and, consequently, better gut eubiosis, especially in young animals under stressful conditions (Gresse et al., 2017). It has recently been shown that a mixture of *B. licheniformis* and *B. subtilis* significantly increases the Simpson’s diversity index in pigs (Wang et al., 2020), and both *B. subtilis* and *B. coagulans* increase the Simpson and Shannon indices in broilers (Li et al., 2019). *B. amyloliquefaciens* has been observed to affect the beta diversity index in post-weaning pigs (Hu et al., 2018). The promotion of the colonization of the gut by beneficial bacteria has also been ascribed to several Bacillus-based probiotics. For instance, *B. subtilis* favors the intestinal colonization of some beneficial bacteria, including *Lactobacillus, Leucobacter, Bifidobacteria, Megasphaera, Coprococcus* and *Prevotella* (Wang et al., 2019, 2021; Ding H. et al., 2021) in nursery and growing pigs. Likewise, in chickens, an increase in *Enterococcus* (Ciurescu et al., 2020; *B. subtilis* ATCC 6051a in broilers), *Lactobacillus* spp. and *Bifidobacterium* spp. was obtained with *B. subtilis* probiotics (Forte et al., 2016; *B. subtilis* PB6 ATCC-PTA 6737 in laying hens). More recently, it has been demonstrated that some strains of Bacillus, including *B. subtilis* could favor the formation of the intestinal biofilm with the production of proteins including TasA, TapA and BslA as well as a mineral scaffold, eDNA and an exopolysaccharide (EPS). This property allows the *B. subtilis* to attach to the gut surfaces, and interact with other bacteria and the host (Arnaouteli et al., 2021). In agreement with the above, the administration of *B. subtilis* to broilers enhanced the B. *subtilis* spp. biofilm formation in the cecum and modulation of the cecal microbiota (Konieczka et al., 2018).

### Immunostimulatory Effects

The beneficial actions of probiotics also favor the development of a reactive immune system of the host, enhancing its mucosal barrier integrity and acting as immune modulators (Roselli et al., 2017).

The immune-modulatory actions ascribed to certain *Bacillus* strains have been described by several studies involving mice. It has been demonstrated that *B. subtilis* strains (*B. subtilis* B10, *B. subtilis* BS02, and *B. subtilis* (natto) B4 spores) promote the activation of macrophages and the induction of pro−inflammatory cytokines (Xu et al., 2012; Huang et al., 2013). In fact, the spore of these *Bacillus* strains can translocate across microfold cells and transfer into Peyer’s patches in which they can activate the dendritic cells and macrophages which can, in turn, stimulate the production of secretory immunoglobulin A (sIgA) by B cells (Duc et al., 2003; Xu et al., 2012). *In vivo* studies in chickens and pigs have demonstrated this capacity of some *Bacillus* strains. In broiler chickens, it has been shown that the local immune system response (proliferation of splenic lymphocytes) was increased by the administration of a mixture of Bacillus probiotics (Lee et al., 2010); *B. coagulans* enhanced the levels of interferon-alpha (IFNα), toll-like receptor 3 (TLR3) and sIgA in the duodenum of broilers (Xu L. et al., 2017). In pigs, similar modulatory effects on pro-inflammatory cytokines have been ascribed to *B. subtilis* ASAG 216 (Jia et al., 2021) and *B. subtilis* (DSM 25841) (Kim et al., 2019).

In addition to the immunomodulatory effect, antioxidant activity has been reported for some *Bacillus* strains. This effect seems to be mediated via the production of antioxidant enzymes which can protect the host from oxidative stress. Oxidative stress is characterized by a high intracellular level of reactive oxygen species (ROS) which can damage proteins, lipids and nucleic acids (Schieber and Chandel, 2014). Of the antioxidant enzymes, *Bacillus* spp. have been reported to produce superoxide dismutase (SOD), glutathione peroxidase (GPx) and glutathione reductase (GR). *B. subtilis* fmbJ (BS fmbJ) increased the level of glutathione (GSH), GR, glutathione peroxidase (GSH-Px), and SOD activity in the serum and livers of broilers, resulting in a reduction in ROS contents in the liver mitochondria (Bai et al., 2017). Similarly, *B. subtilis* natto, *B. licheniformis* and *B. cereus* increased the GPx activity and O_2_- level in the blood, and hepatic catalase and SOD activities in broilers (Gong et al., 2018). Similar observations have been reported by Zhao et al. (2020) regarding the supplementation of *B. licheniformis* H2 stain on broilers in which subclinical necrotic enteritis was induced. The probiotic increased the activity of several antioxidative enzymes (in serum: SOD, CAT, total antioxidant capacity [T-AOC] and GSH; in the ileum: SOD, CAT and T-AOC and in the liver: SOD and CAT).

Overall, the immune-modulatory action enhances the mucosal barrier integrity (Kim et al., 2019; Tang et al., 2019), and the intestinal mucosal structure increases the villus height and the villus height to crypt depth ratio (Ding H. et al., 2021; Fu et al., 2021; Wang et al., 2021). The increase in mucosal permeability is associated with the loss of gut mucosa integrity and can favor the translocation of gastrointestinal pathogens into the systemic circulation of the host, resulting in damage to the liver and sepsis (Rishi et al., 2009). *Bacillus* strains can reduce the pathogen translocation, resulting in less damage to the liver. Recently, aspartate transaminase (AST) and alanine transaminase (ALT), which are two key enzymes involved in the interaction of amino acids with other metabolic intermediates, have been proposed as tissue and liver damage markers. Studies regarding broilers and pigs have demonstrated modulation of AST and ALT by *Bacillus* strains (Wu et al., 2015; Cao et al., 2019; Abdel-Moneim et al., 2020).

### Contribution of Feed Efficiency

A mode of action by which *Bacillus* strains can improve the growth performance of animals could be ascribed to the production of extracellular enzymes, vitamins and peptides which could improve the digestibility of nutrients, indirectly promote growth and modulate the abundance of commensal and beneficial bacteria in the gut. According to Elshaghabee et al. (2017), the majority of the *Bacillus* strains, including *B. coagulans*, *B. subtilis*, *B licheniformis, B. amyloliquefaciens* and *B. cereus*, could produce enzymes capable of degrading carbohydrates (namely α-Amylase, β-Amylase, arabinase, cellulase, chitinase, chitosanase, dextranase and galactanase), peptides (namely aminopeptidase, esterase and serine proteases) and lipids (namely phospholipase C) (Ghani et al., 2013; Elshaghabee et al., 2017). This mode of action has not been fully investigated in monogastric animals; however, studies regarding broilers, ducks and quail have suggested that *Bacillus* strains, including *B. subtilis* and *B. licheniformis*, could improve the amount of digestive enzyme activity in the intestinal content, suggesting enhancement of the feed utilization rate (Rajput et al., 2013; Gong et al., 2018; Li et al., 2019; Abdel-Moneim et al., 2020). Better feed digestion and nutrient absorption capability are also indirectly due to the immunostimulatory effect of *Bacillus* strains as well as controlling oxidative stress which can significantly improve the intestinal morphology of the intestinal tract. In addition, it has been reported that *Bacillus* strains can produce vitamins of the B group, including cobalamin (B12) and inositol (B7) (Mohammed et al., 2014; Tanaka et al., 2014). However, to the Authors’ knowledge, no *in vivo* studies have been carried out in pigs and poultry. Finally, secondary metabolites, including acetic acid and lactic acid, have been reported in *Bacillus* spp. which can contribute to improving the feed efficiency of monogastric species.

## Application of *Bacillus* spp. Probiotic Strains in Livestock Monogastric Animals

The use of probiotics in livestock requires a safety evaluation process which is regulated by specific regulatory rules in Europe and the United States, although no single standard is available (European Union [EU], 2003; FDA, 2015). In Europe, probiotics are evaluated for their safety and efficacy by the scientific panel on additives, and products or substances used in animal feed (FEEDAP Panel) of the European Food Safety Authority (EFSA) which provides an independent opinion in support of the decision of the EU Commission. In the United State, probiotics are generally evaluated and included either on the list of the official drugs, on the list of the Official Publication of the Association of American Feed Control Officials (AAFCO) or on the list of the approved feed additives produced by the Food and Drug Administration (FDA). The risks associated with the inclusion of probiotics in the feed of animals are related to the development of intestinal and systemic infection, the transfer of antibiotic resistant genes, the spread of infectious micro-organisms or noxious compounds to the environment from the animal production system, the production of toxic of harmful metabolites for animals and the hyperstimulation of the immune system of the animals (Bajagai et al., 2016). The main concerns regarding the safety of *Bacillus* strains are associated with their potential production of enterotoxins, the transfer of antibiotic-resistance genes and cytotoxicity against normal cells; two species of *Bacillus*, namely *B. anthracis* and *B. cereus*, are known as pathogens for humans (Cutting, 2011). The safety of the *Bacillus* species in livestock has been extensively reviewed elsewhere (SCAN, 2000; Joerger and Ganguly, 2017). In livestock, the main concerns are for *B. cereus* and *B. licheniformis* since they have been associated with an increase in mastitis in cattle and occasionally associated with bovine toxemia and abortions, respectively (Johnson et al., 1994; Parkinson et al., 1999). To overcome this issue, the full genome (including chromosomes and plasmids) should be sequenced to search for genes coding for enterotoxins and cereulide synthase; the non-functionality of these genes should be verified before allowing the use of *B. cereus* as a probiotic for animals (EFSA Panel on Additives and Products or Substances used in Animal Feed [FEEDAP], 2014).

### Research Analysis Approach

A literature review was carried out in January 2021 to assess and categorize recent scientific contributions carried out on Bacillus probiotics in livestock monogastric species, using the Web of Science and Pubmed databases to identify all articles written in English published between 2011 and 2021, matching the keywords: “Bacillus”; AND “pig” OR “piglet” OR “sow” OR “chicken” OR “broiler” OR “poultry”; AND “performance” OR “health”.

This search resulted in 14,509 articles. Duplicate articles and articles not focusing on the use of *Bacillus* spp. in *in vivo* studies were excluded. In addition, articles reporting research studies conducted only *in vitro* were excluded as were reviews, editorials, book chapters, opinions and non-peer-reviewed articles. After filtering, a total of 131 articles were selected, and were then categorized based on the animal species and phase of production as follows: (1) piglets defined as post-weaning piglets; (2) lactating and gestating sows and (3) broilers.

### Application of *Bacillus* Strains to Post-weaning Piglets

A total of 49 studies were found in the literature regarding the use of *Bacillus* strains in post-weaning pigs. Supplementary Table 1 reports the main results obtained from the literature search.

As frequently reported in the literature, the post-weaning period is one of the most critical phases of the rearing system since a plethora of social, physiological and environmental changes occur and give rise to the so-called “weaning stress” which can predispose the piglets to dysbiosis (Gresse et al., 2019; Trevisi et al., 2021). Piglet dysbiosis is characterized by a loss of microbial diversity and an increase in facultative anerobic bacteria, including *Enterobacteriaceae*, *Proteobacteriaceae* and *Clostridiaceae*, and is then linked to an increase in local intestinal inflammation, and an overall increase in morbidity and mortality (Gresse et al., 2019). With this target, Bacillus probiotics can benefit piglet production, restabilizing the microbial diversity, counteracting the growth of pathogenic bacteria and enhancing the gut barrier function; as a final output, it promotes the health and growth performance of post-weaned pigs. *Bacillus* strains supplied during the immediate post-weaning phase can reduce post-weaning diarrhea and sustain the gut health of piglets.

#### Application of *Bacillus* Probiotic Strains to Control Post-weaning Diarrhea

**FIGURE 2:**
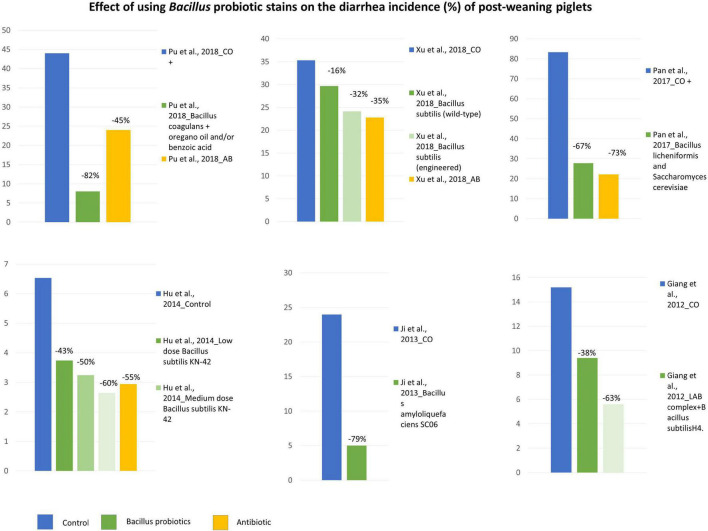
The effect of Bacillus probiotics on the diarrhea incidence (%) of post-weaning piglets as compared with no probiotic or antibiotic supplementation.

The effect of *Bacillus* strains in controlling diarrhea in post-weaning pigs was reported in 16 out of 49 studies (Supplementary Table 1). Except for a few studies, namely Altmeyer et al. (2014), Zhou et al. (2015), and Canning et al. (2017), the majority of studies agreed that the use of *Bacillus* strains could favor controlling post-weaning diarrhea, even in piglets challenged with pathogens (Supplementary Table 1). The majority of the studies selected used the percentage of diarrhea incidence, calculated as the number of (diarrhea piglets × diarrhea days/the number of piglets × test days) × 100, as a parameter for evaluating the effect of *Bacillus* strains in controlling post-weaning diarrhea. A summary of the results of the studies reporting the diarrhea incidence% is shown in Figure 2. It can be observed that, in all the studies selected, supplementation with *Bacillus* strains reduced the diarrhea indices (%) by at least 30% [except for the study of Xu J. et al. (2017)] to values ranging from 79 to 82% (Ji et al., 2013; Pu et al., 2018) as compared with the untreated control groups (Figure 2). Furthermore, in these same studies, it can be observed that the reduction in the incidence of diarrhea% achieved using *Bacillus* strains was equal (Hu et al., 2014; Pan et al., 2017; Xu J. et al., 2017) or greater (Pu et al., 2018) than the diarrhea% achieved using antibiotics. Similarly, using different parameters related to the occurrence of diarrhea, including the fecal score scale and the diarrhea score, Tan et al. (2020; *B subtilis* PB6 + essential oils) and Zong et al. (2019; *B. licheniformis* + ZnO at 1125 mg/kg) demonstrated that probiotic supplementation with *Bacillus* strains contributed to reducing the diarrhea of post-weaning pigs to the same extent as with the use of antibiotics. Therefore, the effect of the specific *Bacillus* probiotic strains is difficult to determine since each study tested different strains. Taken together, the data collected indicated that the *Bacillus* strains generally controlled the occurrence of post-weaning diarrhea in piglets.

#### Application of *Bacillus* Probiotic Strains to Improve the Gut Health of Piglets

The overall improvement of the growth performance of post-weaning piglets fed with Bacillus probiotics can be ascribed to the proven beneficial effect that some *Bacillus* strains exert on gut health, reducing the colonization of the gut by pathogens, modulating the commensal bacteria, enhancing the gut barrier function and the mucosal morphology, and stimulating the mucosal immune system. Table 1 shows some of the beneficial and antibiotic-comparable effects that *Bacillus* strains may have on the gut health of piglets. Villus height and crypt depth are known to play a crucial role in nutrient absorption and digestion. *Bacillus* strains have demonstrated a beneficial effect on villus height and crypt depth; an increase in the villus height/crypt depth ratio with a value which is comparable to the use of antibiotics was demonstrated by Fu et al. (2021; *B. coagulan*s), Wang et al. (2020; *B. licheniformis-B. subtilis mixture*) and Choi et al. (2011; a mixture of Lactobacillus + *B. subtilis* CH201/DSM 5750 and *B. licheniformis* CH200/DSM 5749 or mixture of *Saccharomyces cerevisiae* + *Lactobacillus* + *B. subtilis* CH201/DSM 5750 and *B. licheniformis* CH200/DSM 574). Moreover, the increase in the level of occludin protein in the jejunal mucosa, villus height and villus height to crypt depth ratio in the duodenum, jejunum and ileum was observed by Pan et al. (2017; *B licheniformis* and *Saccharomyces cerevisiae*). In contrast, no effect on the intestinal morphology was observed in two other studies (Luise et al., 2019; Zong et al., 2019). Differences in the timing of sampling, as well as the sanitary status of the animals, can strongly affect the gut morphology measurements.

**TABLE 1:** The effect of Bacillus probiotics on the intestinal mucosa and gut microbiota of post-weaning piglets as compared with antibiotic use.

Probiotic	Antibiotic group	Effect^1^	References
**Intestinal mucosa**			
*B. coagulans*	20 mg/kg colistin sulfate and 40 mg/kg bacitracin zinc	= villus height and crypt depth > villus height/crypt depth than CO and = to AB	Fu et al., 2021
*B. licheniformis* + *B. subtilis*	0.04 kg t^–1^ virginiamycin, 0.2 kg t^–1^ colistin and 3,000 mg kg^–1^ zinc oxide	< jejunum crypt depth > ileum villus height, and the jejunum and ileum villus height to crypt depth ratio > expression level of E-cadherin in the colon and proinflammatory cytokines, and TLR-4 in ileum and colon	Wang et al., 2020
*B. licheniformis* + ZnO at 1,125 mg/kg	100 mg olaquindox/kg, 20 mg colistin sulfate/kg, and 50 mg kitasamycin/kg and ZnO (2250 mg Zn from ZnO/kg)	= the villus height, crypt depth and villus height/crypt depth ratio = in the gene expression	Zong et al., 2019
*B. amyloliquefaciens* DSM25840 or *B. subtilis* DSM25841	yes, 1 g colistin/kg of feed (AB).	> several gene sets related to immune response, including gene sets involved in stimulus detection and in adaptive immune response capability (B and T cell lymphocyte activation) as AB	Luise et al., 2019
Mixture of *C. butyricum* CGMCC, *B. subtilis* CGMCC and *B. licheniformis* CGMCC	100 mg colistin sulfate per kg,	> ileum villus structure than CO and AB jejunum villus morphologies = CO Intestinal apoptotic cells = to AB and > CO	Cao et al., 2019
*B. coagulans* + oregano oil and/or benzoic acid	20 g/t colistin sulfate + 40 g/t baci- tracin zinc	< TNFalpha and IL-1B compared with the CO and = AB in the jejunum mucosa; > sIgA than CO and = AB	Pu et al., 2018
*B. licheniformis* and *S. cerevisiae*	100 mg/kg zinc bacitracin (10%) 50 mg/kg colistin sulfate (10%), and 100 mg/kg olaquindox (5%)	> occludin protein in the jejunal mucosa > villus height and villus height to crypt depth ratio in the duodenum, jejunum and ileum.	Pan et al., 2017
*E. faecium* + *B. subtilis*, *and B. licheniformis*	55 mg/kg carbadox (antibi-	> crypt depth in the jejunum = crypt depth in the duodenum = gut health compared with CO and AB groups	Walsh et al., 2012
Mixture of *Lactobacillus* + *B. subtilis* CH201/DSM 5750 and *B. licheniformis* CH200/DSM 5749 or mixture of *S. cerevisiae* + *Lactobacillus* + *B. subtilis* CH201/DSM 5750 and *B. licheniformis* CH200/DSM 5749	0.10% chlortetracycline and Aurofac200C containing 100 g of chlortetracychne/Kg	> villus height at the jejunum and ileum, > villus height:crypt depth at the ileum compared with CO and = AB	Choi et al., 2011
**Microbiota**			
*B. licheniformis* + *B. subtilis* mixture	0.04 kg t^–1^ virginiamycin, 0.2 kg t^–1^ colistin and 3,000 mg kg^–1^ zinc oxide	> Simpson’s diversity index > Bacteroidetes and *Ruminococcaceae* < *Blautia* and *Clostridium*.	Wang et al., 2020
Mixture of *C. butyricum* CGMCC, *B. subtili*s CGMCC and *B. licheniformis* CGMCC	100 mg colistin sulfate per kg,	= alpha diversity > microbial metabolism for amino acids, oxidative phosphorylation, amino acid-related enzymes, DNA repair, replication and recombination proteins, and secretion systems	Cao et al., 2019
*B. amyloliquefaciens* cells (China Center For Type Culture Collection, No: M2012280)	150 mg/Kg aureomycin, G2	= alpha diversity ≠ in β-diversity = relative abundance at the phylum and family levels	Hu et al., 2018
*B. coagulans* + oregano oil and/or benzoic acid	20 g/t colistin sulfate + 40 g/t baci- tracin zinc	> *Lactobacillus* and *Bifidobacterium* compared with CO and = to AB	Pu et al., 2018
*B. licheniformis* and *S. cerevisiae*	100 mg/kg zinc bacitracin (10%) 50 mg/kg colistin sulfate (10%), and 100 mg/kg olaquindox (5%)	< cecal *E. coli* count compared with CO; > cecal *Lactobacillus* count compared with the CO and AB groups	Pan et al., 2017
Mixture of *Lactobacillus* + *B. subtilis* CH201/DSM 5750 and *B. licheniformis* CH200/DSM 5749 or mixture of *S. cerevisiae* + *Lactobacillus* + *B. subtilis* CH201/DSM 5750 and *B. licheniformis* CH200/DSM 5749	0.10% chlortetracy- cline and Aurofac200C containing 100 g of chlortetra- cychne/Kg	Exp1: < *Clostridium* spp. compared with CO	Choi et al., 2011

*Effect^1:^ CO = negative control group; AB = antibiotic group.*

The effect of *Bacillus* strains on the intestinal microbiota of post-weaning piglets has still not been entirely depicted as results vary depending on the study, *Bacillus* strains and their site in the gut. Currently, the use of the next-generation sequencing (NGS) technique allowed more in-depth investigation and description of the eubiosis among the intestinal bacteria; the use of alpha and beta indices have been widely applied for this purpose. The alpha diversity indices (Chao1, Shannon and Simpson indices), represent the diversity of species within a given sample and, in post-weaning piglets, a decrease in their values has been associated with dysbiosis (Gresse et al., 2017). Hu et al. (2018), Cao et al. (2019), and Luise et al. (2019), and have reported no effect of Bacillus probiotic supplementation on the alpha diversity indices while Wang et al. (2020) observed a decrease in the Simpson’s diversity index. It is however, clear that Bacillus probiotics can affect the abundance of specific taxa. In the early literature, the *Bacillus* strains were known to affect the abundance of Lactobacilli, *E. coli* and Clostridium (Choi et al., 2011; Pan et al., 2017; Pu et al., 2018). It has currently been demonstrated that the abundance of taxa which have been less studied, but are still relevant, including Bacteroidetes, *Ruminococcaceae*, *Faecaliumbacterium* (Wang et al., 2020; Jia et al., 2021), *Leucobacter*, *Cupriavidus* and *Coprococcus* (Ding H. et al., 2021), can also be affected by the supplementation of *Bacillus* strains. Overall, these bacteria are known to produce SCFAs via the fermentation of indigestible fiber; in fact, an increase in SCFAs due to the supplementation of *Bacillus* strains has recently been proven by several studies (Dumitru et al., 2020; Ding H. et al., 2021; Ding S. et al., 2021). The availability of a correct concentration of SCFAs in the gut is important to sustain gut integrity and the immune function. Therefore, this should be considered in the framework of the immunomodulatory effects exerted by the *Bacillus* strains. Moreover, the direct and indirect interactions of bacteria with immune and non-immune cells lead to increasing cell activation and proliferation (He et al., 2020), cytokine production (Wang et al., 2020; Jia et al., 2021) and tight junction improvement (Pan et al., 2017; Tang et al., 2019) in post-weaning piglets fed with Bacillus probiotics (Supplementary Table 1).

#### Application of *Bacillus* Probiotic Strains to Improve the Growth Performance and Feed Efficiency of Piglets

A vast body of literature concerns the capacities of different *Bacillus* strains to enhance productive parameters in piglets (Supplementary Table 1). Interestingly, 14 out of 49 studies investigated the effect of *Bacillus* strains on performance as compared to the use of antibiotics. Beneficial effects regarding average daily gain (ADG), having comparable values between Bacillus probiotics and antibiotics, were observed in several studies, including Li et al. (2021; *B. subtilis*), Hu et al. (2014; *B. subtilis*), Fu et al. (2021; *B. coagulan*s), Cao et al. (2019; a probiotic mixture of *Bacillus* and *Clostridium* strains), Pan et al. (2017; *B. licheniformis* and *S. cerevisiae)* and Choi et al. (2011; *Lactobacillus* + *B. subtilis CH201/DSM 5750 and B. licheniformis CH200/DSM 5749* or mixture of *Saccharomyces cerevisiae* + *Lactobacillus* + *B. subtilis CH201/DSM 5750* and *B. licheniformis CH200/DSM 5749*). In contrast, when considering the studies selected, fewer univocal beneficial effects were demonstrated for the average daily feed intake (ADFI), the feed to gain (F:G) ratio and the gain to feed (G:F) ratio parameters when probiotics were compared to antibiotics (Supplementary Table 1). The growth-promoting effect, as well as amelioration of the feed efficiency, can be associated with improvement in the apparent total tract digestibility (ATTP) of dry matter (DM), gross energy (GE), crude protein (CP) and fat in pigs fed probiotics as reported by Lan et al. (2016; multi-microbe probiotics containing *B. coagulance, B. lichenformis, B. subtilis* and *Clostridium butyricum*), Cai et al. (2015; multi-strain probiotics including 1 strain of *B. subtilis* and 2 strains of *B. amyloliquefaciens*), Choi et al. (2011; multi-microbe probiotic products containing *B. subtilis*), Kunavue and Lien (2012; multi-microbe probiotic products containing *L. acidophilus, B. lactis, B. subtilis* and *B. natto*) and Jørgensen et al. (2016; *B. licheniformis* (DSM 5749) and *B. subtilis* (DSM 5750). In fact, as previously reported, Bacillus is known for its ability to produce various digestive enzymes, including amylase, protease, phosphatase and fiber-digesting enzymes, which may improve the digestibility of the feed. Moreover, better intestinal absorption and secretion activity have also been demonstrated by an increase in the intestinal expression of gene sets related to anion, cation, sodium and potassium channel activation by the supplementation of *B. subtilis* (Luise et al., 2019).

### Application of *Bacillus* Probiotic Strains in Sows

A total of 41 studies were found in the literature regarding the use of *Bacillus* strains in gestating and lactating sows. Although there are a relatively low number of studies, the application of *Bacillus* spp. in sows could be very relevant for improving the efficiency of sows in the production system and also for the contribution that sows have in producing robust piglets. Bacillus supplied during the late gestation and lactation phases can improve sow reproductive performance, gut environment, and blood biochemical and immunological indices.

#### Application of *Bacillus* Probiotic Strains for Improving Sow Body Weight and Fertility

During late gestation and lactation, sows are under increased metabolic burdens which are not covered by an adequate nutrient uptake since sows reduce their voluntary feed intake. According to the literature selected, the supplementation of *Bacillus* strains, during late gestation and lactation, contributed to improving the feed intake of the sows, ensuring a higher quantity of nutrients available, resulting in a reduction in the significant loss of body weight (BW) and backfat thickness, especially during lactation. These beneficial effects have been ascribed to *B. subtilis* supplementation alone (Kritas et al., 2015; Menegat et al., 2019), in combination with other probiotics including *B. licheniformis* (Alexopoulos et al., 2004) or *Lactobacillus acidophilus* (Jeong et al., 2015), or in combination with other feed additives (essential oils) and nutrients (Cr and glucose) (Menegat et al., 2018; Nguyen et al., 2018). Similar beneficial effects on feed intake and BW have also been ascribed to *B. cereus* var. toyoi (CNCM I-1012/NCIMB 40112 and CIP 5832) (Alexopoulos et al., 2001; Taras et al., 2005) and to *B. mesentericus* in combination with *Clostridium butyricum* and *Enterococcus faecalis* strain T-110 (Hayakawa et al., 2016; Inatomi et al., 2017; Tsukahara et al., 2018).

Another relevant beneficial effect of the use of Bacillus probiotics is related to a reduction in the sow weaning-estrus interval which is recognized as an important parameter for sow efficiency. In fact, reduction in the weaning-to-estrus interval is strictly related to the number of litters produced per sows per year. In addition, intervals of 2–4 days result in higher litter sizes while litter size decreased progressively for sows with an interval of 5, 6, and 7 days (Leman, 1990). A reduction in the sow weaning-estrus interval has been reported for *B. subtilis* C-3102 (Kritas et al., 2015) while, for the probiotic mixture composed of *B. mesentericus* TO-A, *Clostridium butyricum* TO-A and *Enterococcus faecalis* T-110, studies have reported contrasting results. In fact, the studies of Inatomi et al. (2017) and Tsukahara et al. (2018) pointed out that this probiotic mixture significantly reduced the weaning-estrus interval while, in contrast to these studies, the study of Hayakawa et al. (2016) reported that the recurrence of estrus tended to be longer in the group treated with a probiotic, but the ratio of return to estrus was significantly increased. Finally, the administration of a mixture of *B. subtilis* and *Lactobacillus acidophilus* during late gestation and lactation seemed not to have affected the weaning-to-estrus interval in the study of Jeong et al. (2015).

Less studied, but noteworthy, is the effect that *Bacillus* strains may have on placental efficiency (ratio of fetal weight to placental weight). According to the study of Gu et al. (2019a), the symbiotic mixture of isomaltooligosaccharide with *B. subtilis* and/or *B. licheniform* during late gestation significantly improved placental efficiency and the growth hormone concentration in umbilical venous serum resulting in improving piglet birth BW. The mode of action was not completely clarified but the authors hypothesized that the higher placental efficiency could have been due to the regulation of the excessive decomposition of fat, which could damage the placenta, and a reduction in placental antioxidant capacity by the probiotics (Gu et al., 2019a). Furthermore, a recent study of Zhou et al. (2020) suggested that the inclusion of *B. subtilis* ANSB01G in the gilt diet could alleviate the adverse effect of dietary mycotoxin on the uterus (reduced lesions) and the apoptosis-related proteins in the uterus, ovary and mammary glands.

In summary, according to the data collected, it could be suggested that Bacillus probiotics could improve sow fertility, depending on the *Bacillus* strain; however, more data are needed to confirm the effect of the specific strains. This beneficial effect of Bacillus probiotics on sow fertility could be ascribed to the overall contribution which probiotics may have on the sow body condition which is highly correlated with sow fertility (Dial et al., 1992).

#### Application of *Bacillus* Probiotic Strains for Controlling the Intestinal Microbial Balance of Sows

The supplementation of sows with *Bacillus* strains may also modulate their intestinal microbial ecosystem and, in turn, affect the sow metabolism and immunity as well as the early life colonization of the gut microbiota of their offspring. Few studies have investigated this aspect; only one study investigated the effect of Bacillus probiotics on the sow microbiota during gestation. In that study, the authors showed that the administration of *B. cereus* var. toyoi to sows during gestation did not change the presence of pathogenic bacteria in the feces of the sows (Schierack et al., 2009). However, it is known that the microbiota of sows is more stable than that of young animals, such as piglets. The majority of the studies investigated the effect of Bacillus probiotic supplementation during late gestation and lactation, especially to evaluate the effect on their offspring. For instance, the diversity of the gut microbial community decreased when sows were fed *B. subtilis* PB6 but it favored the colonization of the gut with beneficial bacteria, including *Gemmatimonadete*, *Acidobacteria* and *Ruminococcaceae*_UCG-013 cc (Zhang et al., 2020). In the same way, dietary *B. subtilis* supplementation increased the abundance of the *Lactobacillus* species and reduced the abundance of *Clostridium perfringens* and *E. coli* in the colon of sows (Baker et al., 2013). Piglets born to and nursed by *B. subtilis* C-3102 probiotic-fed sows had a similar fecal microbial population to each other with an increasing abundance of *B. subtilis* C-3102 and total *Bacillus* spp. in the preweaning period (Menegat et al., 2019). An increase in Bacillus spores in the intestinal content of piglets born to Bacillus probiotic-fed sows was also confirmed by Poulsen et al. (2018). In summary, the main strain tested in the modulation of the sow microbiota was *B. subtilis*; the studies selected evidenced a moderate modulation in the sow microbiota which is, in general, more difficult to modify. The effect of the modulation of piglet early life colonization by sows fed Bacillus is still to be investigated, although the increase in Bacillus colonization in the piglet gut may be a promising perspective.

#### Application of *Bacillus* Probiotic Strains for Controlling Sow Oxidative Stress and Immunity Parameters

The increased metabolic burdens which occur during late gestation and lactation, in addition to reducing voluntary feed intake, also cause elevated systemic oxidative stress to the sow. Benefits in reducing oxidative stress have been reported with the use of Bacillus probiotics in sows but only in a limited number of studies. Zhang et al. (2020) reported that the administration of *B. subtilis* PB6 reduced the oxidative stress in sows, notably by a decrease in malondialdehyde (MDA) and an increase in serum T-AOC at parturition and serum catalase (CAT) concentrations on day 21 of lactation. The decrease in blood insulin, cortisol and glucose levels has also been observed in sows fed a diet supplemented with feed additives, including *Bacillus* strains (Nguyen et al., 2018: *B. subtilis*; Gu et al., 2019a: *B subtilis* + *B licheniformis*; Zhang et al., 2020: *B subtilis* PB6). However, since the probiotics in these latter studies were used in combination with other nutrients/additives, the clear effect of the Bacillus is difficult to identify.

The effect of the administration of *Bacillus* strains on sows with respect to their immunity has been poorly investigated. However, it is known that pregnancy can suppress the immunological functions contributing to the susceptibility to pathogen infection. The administration of *B. cereus* var. toyoi can improve the immune function of pregnant sows by altering the proliferative response of lymphocytes, notably by an increase in CD21 + lymphocytes which are predominantly responsive to bacterial antigens (Schierack et al., 2009). By the same token, an increase in blood lymphocyte percentage at weaning was observed by supplementing the sow diet with *B. subtilis* (1.2 × 10^7^ cfu/g) and *L. acidophilus* (1.15 × 10^6^ cfu/g) during late gestation and lactation (Jeong et al., 2015). More recently, a probiotic mixture including *B. mesentericus* increased the specific antibody titer against porcine epidemic diarrhea (PED) virus in both vaccinated and unvaccinated pregnant sows (Inatomi et al., 2017; Tsukahara et al., 2018). Again, the lack of available data did not allow the authors to draw any conclusions regarding the effect of the specific probiotic strain on oxidative stress and the immune parameters of the sows; additional investigations are needed.

#### Application of *Bacillus* Probiotic Strains for Improving Colostrum Milk Quality and Litter Performance

Overall, the increase in the feed intake coupled with control of the oxidative stress and immune response ascribed to the Bacillus probiotic also contributed to improving the quality of colostrum and milk. For example, the additive mixture including *B. mesentericus* tested by Inatomi et al. (2017) and Tsukahara et al. (2018) not only increased the blood PED-specific immunoglobulin G (IgG) levels in vaccinated and unvaccinated sows but also significantly increased the concentration of total IgA and IgG in the milk (Inatomi et al., 2017), and contributed to improving the quantity of proteins in the milk (Inatomi et al., 2017). No effect on colostrum and milk protein, and fat composition was observed by Gu et al. (2019b) while, according to Inatomi et al. (2017), an increase in total IgA and IgG was observed by Gu et al. (2019b) in the sows fed *Bacillus* spp. + isomaltooligosaccharide. Similar beneficial effects have been observed in the quality of colostrum and milk of sows supplemented with *B. cereus* CIP 5832 or *B. licheniformis* DSM 5749 plus *B. subtilis* DSM 5750. In fact, in both cases, the probiotic supplement contributed to inhibiting the reduction of fat and protein milk content which generally occurs from day 3 to day 14 postpartum (Alexopoulos et al., 2001, 2004).

The increase in colostrum and milk quantity and quality, together with the generally better health condition of the sows supplemented with *Bacillus* strains during late gestation and lactation may contribute to improved sow reproduction performance in terms of a higher number of piglets born, reduction in stillborn piglets, lower piglet pre-weaning mortality and higher weaning piglet BW as has been reported in several studies (Alexopoulos et al., 2004; Taras et al., 2005, 2007; Baker et al., 2013; Jeong et al., 2015; Kritas et al., 2015; Hayakawa et al., 2016; Inatomi et al., 2017; Nguyen et al., 2018; Tsukahara et al., 2018; Zhang et al., 2020). A summary of the beneficial effect of probiotic administration regarding sows on their reproductive performance is shown in Table 2. For the most part, the supplementation of sows has been tested regarding the duration of late gestation and lactation and, regardless of the type of *Bacillus* strain used, the main beneficial results observed were an increase in the BW and ADG of the piglets during the suckling period, and reduced mortality. Therefore, although the results regarding the effect of *Bacillus* spp. in modulating sow immunity, oxidative stress and microbial balance have been poorly supported by published data, the more consistent conclusion regarding the beneficial effect of improving sow reproductive performance has suggested an overall improvement in the health of the sows and merits additional investigation.

**TABLE 2:** The effect of Bacillus probiotics on sow productive performance.

Probiotic	Period of administration	Effect on the litter	References
Mixture of *B. subtilis* (2.0 × 10^11^ cfu g-1) + isomaltooligosaccharide or *B. subtilis* (2.0 × 10^11^ cfu g-1) and *B. licheniformis* (2.0 × 10^11^ cfu g-1) *B. licheniformis* (2.0 × 10^11^ cfu g-1) + isomaltooligosaccharide	Late gestation to weaning	> BW at weaning and ADG during suckling	Zhang et al., 2020
Mixture of *B. mesentericus* TO-A (1 × 10^6^ cfu/g) + *C. butyricum* TO-A (1 × 10^6^ cfu/g) + *E. faecalis* T-110 (1 × 10^8^ cfu/g)	Four weeks prior to farrowing to one-week post-farrowing	> BW at birth and < mortality percentage during the first 21 days of suckling	Tsukahara et al., 2018
Mixture of 0.03% of *B. subtilis* + essential oil (0.02%), Cr (0.05%) and glucose (0.18%)	From d 107 of gestation until farrowing,	> ADG during suckling	Nguyen et al., 2018
*B. subtilis* C-3102	From d 30 of gestation until farrowing and during lactation	= ADG and BW until weaning < ADG and BW in the post-weaning phase	Menegat et al., 2018
Mixture of *B. mesentericus, C. butyricum*, and *E. faecalis*	Six weeks before farrowing to one-week after farrowing	> BW at weaning and a < mortality percentage	Inatomi et al., 2017
Mixture of *B. mesentericus* TO-A (1 × 10^8^ cfu/g) + *C. butyricum* TO-A (1 × 10^8^ cfu/g) + *E. faecalis* T-110 (1 × 10^9^ cfu/g)	Late gestation and lactation to sows and to piglets from the age of 7 days to weaning	> BW at weaning, > FCR and < diarrhea	Hayakawa et al., 2016
Mixture of *B. subtilis* (1.2 × 10^7^ cfu/g) + *L. acidophilus* (1.15 × 10^6^ cfu/g).	Late gestation to weaning	> BW at birth	Jeong et al., 2015
*B. subtilis* spores	Gestation and lactation	> number of total and alive birth piglets; > BW, ADG and litter size at weaning	Baker et al., 2013
*B. subtilis* C-3102	Gestation and lactation	> BW at weaning	Kritas et al., 2015
Mixture of *E. faecium* NCIMB 10415 + *B. cereus* var. toyoi	Gestation and lactation and to piglets pre and post weaning	< post-weaning diarrhea index; > G:F ratio post-weaning	Taras et al., 2007
*B. cereus* var. toyoi	Late gestation to weaning	> ADG, G:F and < incidence of liquid feces and post-weaning diarrhea	Taras et al., 2005
Mixture of *B. licheniformis* + *B. subtilis* spores	Late gestation to weaning	> BW at weaning and < diarrhea score and pre-weaning mortality	Alexopoulos et al., 2004
*B. cereus* CIP 5832	Late gestation to weaning	> BW, ADG and FCR < mortality	Alexopoulos et al., 2001

### Application of *Bacillus* Probiotic Strains to Broilers

The avian industry has always been characterized by the need for maximizing the feed conversion and growth rates and, at the same time, for controlling the gastrointestinal disorders associated with the development of bacterial infections caused by the following bacteria: Staphylococcus, Pseudomonas, Escherichia, Salmonella, Streptococcus, Campylobacter, Yersinia and Clostridium (Agyare et al., 2018). In broilers, as has already been reported in pigs, gut dysbiosis mainly occurs during the first weeks of life since the immune system is not fully developed until the seventh-eighth week of life (Song et al., 2021) and the gut microbial ecosystem is not stable (Ohimain and Ofongo, 2012). Therefore, Bacillus probiotics may contribute to stabilizing the gut microbiota and stimulating the gut immune function, improving broiler digestion and growth performance. A total of 41 studies were found in the literature and were selected for this systematic review (Supplementary Table 2). The main results derived from the application of Bacillus probiotics in terms of health and survival, gut health, growth performance and feed efficiency are summarized in the following sections.

#### Application of *Bacillus* Probiotic Strains to Improve the Health and Survival of Broilers

Minimizing mortality in the poultry flock is crucial for making a profit in the poultry industry, so much so that, according to European standards (European Union [EU], 2007) when the daily cumulative mortality rates are too high, farmers are asked to reduce the number of broiler chicks in the next cycle. According to the literature selected, the use of *Bacillus* strains showed promising results in reducing broiler mortality by approximately 6% as reported by Teo and Tan (2006) and Whelan et al. (2019), resulting in mortality percentages similar to those obtained using antibiotics. For example, the percentage of mortality of birds infected with a pathogenic strain of *E. coli* was reduced from 14% (negative control) to 6% in the antibiotic-treated groups and 8% in the *B. subtilis* PB6-treated group (Teo and Tan, 2006). In the same way, the mortality of birds challenged with *Eimeria maxima* oocysts was reduced by 6.5% when supplemented with *Bacillus subtilis* DSM 32315 and by 9.1% when treated with Narasin (Whelan et al., 2019). According to Whelan et al. (2019), the beneficial effect of *Bacillus* subtilis DSM 32315 in reducing bird morality could be related to the contribution of the probiotic in improving gut eubiosis and inhibiting the colonization of the gut with potentially pathogenic bacteria, including *C. perfringens*. In fact, the authors observed a reduction in the abundance of *C. perfringens* in the ileum of the birds in the probiotic and antibiotic groups as compared with the control group (Whelan et al., 2019).

#### Effect of *Bacillus* Probiotic Strains on Gut Health, the Microbiota and Immune Functions of Broilers

The effect of *Bacillus* strains on gut health and the microbial profile was investigated in 11 and 19 of the 41 studies, respectively. Considering the 19 studies investigating the gut microbiota, only 1 study (Blajman et al., 2017) found no difference in the microbial population profile between the control and the Bacillus-supplemented broilers. The majority of the studies evidenced the capacity of the *Bacillus* strains tested to influence the gut microbial profile and to promote beneficial bacteria in a better way as compared to that which an antibiotic administration could have. An increase in the abundance of *Lactobacillus* spp. within the intestinal tract was seen with the use of a probiotic mixture containing Bacillus as compared with one containing avilamycin (Kazemi et al., 2019), and with the use of *B. subtilis* PB6 when compared with birds included in the negative control (maduramicin ammonium) and antibiotic groups (bacitracin zinc + colistin sulfate) (Teo and Tan, 2006). Similarly, *B. subtilis* DSM 32315 favored the colonization of the ileum and the cecum by *Lactobacillus johnsonii*, known as beneficial bacteria, and decreased the abundance of the *Lachnospiraceae* and *Ruminococcaceae* families as compared with the control and the Narasin groups (Whelan et al., 2019; Trela et al., 2020). Both the *Lachnospiraceae* and *Ruminococcaceae* families are generally present in the poultry gut microbiota; however, an increase in *Ruminococcaceae* has previously been reported to favor *Eimeria* spp. infections (Wu et al., 2014). In addition, a decrease of 3–10% in *Clostridium*, *Coliforms* and *Campylobacter jejuni* has been observed in groups of animals supplemented with Bacillus probiotics (Sinol Sen et al., 2011; Kazemi et al., 2019). The combined use of *B. licheniformis* and salinomycin in the diets of broilers also promoted modulation of the gut environment since a reduction in pH in the cecal digesta reduced the abundance of *Enterobacteriaceae*, and promoted the abundance of *Bacillaceae* communities in the jejunum and *Clostridiaceae* in the cecum (Trela et al., 2020). The modulatory effect of *Bacillus* strains on the gut microbiota was expected as it has been recognized that some *Bacillus* strains could colonize the intestinal tracts of broilers, forming a mucosal biofilm and, therefore, be part of the mucosa-associated bacterial communities (Konieczka et al., 2018). The interaction between the *Bacillus* strains and the intestinal mucosa could also promote a local intestinal innate immune response in broilers derived from the host-bacteria cross-talk. For instance, according to Kazemi et al. (2019; probiotic mixture containing *B. subtilis*) and Lourenco et al. (2012; *B. subtilis* DSM 17299), bacillus supplementation led to an increase in the number of Goblet cells (+ 23% and + 19%, respectively) in the intestinal tracts of broilers, and a reduction in the severity of the infiltration by inflammatory cells in the jejunum was attributed to supplementation with *B. subtilis* DSM 17299 (Koli et al., 2018). In addition, the supplementation of *B. subtilis* DSM 17299 significantly improved the number of CD4 + in the ileum and cecum (+ 30% and + 50% as compared with the control group, respectively) of 7-day old broilers (Lourenco et al., 2012). In an interesting study conducted by Lee et al. (2010), the effect of 9 different strains of *B. subtilis* was investigated regarding the immunological parameters, including the expression of T cell surface markers (cluster of differentiation (CD) CD3, CD4, CD8, T-cell receptor (TCR) TCR1, TCR2) in the intestinal lymphocytes. They are considered to be the primary immune effector cells in the gut since they can recognize pathogens and promote the release of antimicrobial compounds, cytokines and chemokines in order to activate the adaptive immune response. According to the results of Lee et al. (2010), the most promising *Bacillus* strains capable of increasing the number of T cells expressing CD3, CD4, CD8, TCR1, TCR2 were 15AP4, Bs27 or Avicor. Furthermore, the authors observed the increased, decreased or unchanged expression of different intestinal cytokines as compared with controls, depending on the strains used (Lee et al., 2010). Unfortunately, no more recent studies aimed at comparing the effect of different probiotic strains on gut immunity, such as that by Lee et al. (2010), were found for this systematic review; however, according to the studies available, the dietary supplementation of *Bacillus* strains at an early age would appear to enhance broiler immune competence.

**FIGURE 3:**
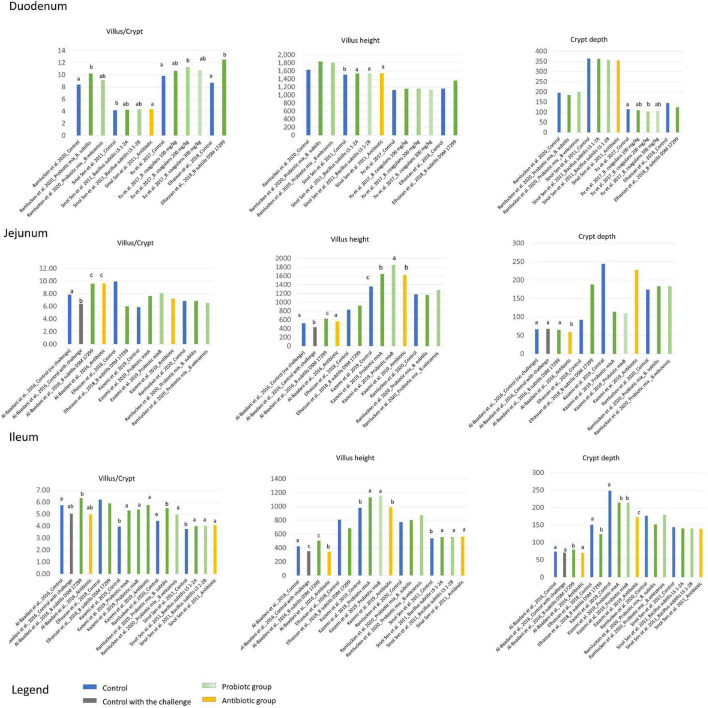
The effect of Bacillus probiotics on the villus/crypt ratio, villus height and crypt depth in the duodenum, jejunum and ileum of broilers as compared with no probiotic or antibiotic supplementation. The letters represent significant differences among the groups within the studies.

To ensure good gut health, the proper digestion and absorption of nutrients are also relevant. Gut functionality is strictly related to the surface of the epithelium and, therefore, the villus height and crypt depth have been extensively investigated as markers for evaluating the potential effect of *Bacillus* strains as probiotics and, therefore, as a potential tool for improving gut health. Figure 3 summarizes the results reported in the studies included in the present review which investigated the effect of *Bacillus* strains on villus height, crypt depth and the villus/crypt ratio in the duodenum, jejunum and ileum of broilers. Overall, the effect appears to depend on the intestinal tract and the *Bacillus* strains; however, more consistent results can be observed in the ileum in which, independent of the strains, the Bacillus supplementation was able to increase the villus height as compared with the controls (Al-Baadani et al., 2016: + 19%; Kazemi et al., 2019: + 15% and + 17%; Sinol Sen et al., 2011: + 4%), and the villus/crypt ratio (Al-Baadani et al., 2016: + 10%; Kazemi et al., 2019: + 34% and + 36%; Sinol Sen et al., 2011: + 6%), having comparable results in the various antibiotic groups (Sinol Sen et al., 2011; Al-Baadani et al., 2016; Kazemi et al., 2019). These promising results observed in terms of gut morphological parameters could, in turn, promote the digestion of nutrients and, as a final outcome, the growth performance of broilers which will be described in the next paragraph.

Under stressful conditions, namely early age, leaky gut or disease, broilers undergo an increase in oxidative stress which represents an imbalance between free radicals and antioxidants in the body. Similar and comparable results between the use of *Bacillus* strains and antibiotics have also been observed in the regulation of oxidative stress in broilers. According to Kazemi et al. (2019), supplementation with Bacillus probiotics or antibiotics reduced the malondialdehyde levels (as a marker of lipid peroxidation) in meat. Zhang et al. (2021) showed that supplementation with *B. coagulans* significantly increased catalase (by 44%), SOD (by 8.8%) and GPx (by 58%) levels, and reduced the malondialdehyde level (by 21%), improving the antioxidant capacity overall (Zhang et al., 2021). Additional studies have confirmed the same promising results of *Bacillus subtilis* fmbJ (Bai et al., 2017), *B. subtilis* ATCC PTA-6737 (Abudabos et al., 2017) and *B. licheniformis* H2 (Zhao et al., 2020).

According to the data collected, the use of bacillus probiotics for the improvement of broiler gut health in terms of morphological parameters, favorable modulation of the gut microbiota and local adaptative immune response, and the reduction of the systemic oxidative stress of broilers gives promising results. These positive and antibiotic-comparable effects derived from the administration of Bacillus probiotics could be due to the different modes of action reported previously.

#### Application of *Bacillus* Probiotic Strains to Improve Growth Performance and the Feed Efficiency of Broilers

A summary of the *Bacillus* probiotic strains, period of administration and main effects on broiler growth performance is reported in Supplementary Table 2. On the whole, it could be observed that Bacillus probiotics significantly contributed to improving broiler performance in terms of body weight gain (BWG) and ADG compared to the control group fed the basal diet. Notably, of the studies selected, 8 out of 35 studies included a group in which antibiotics were used as the reference control group; therefore, this allowed comparing the effect of *Bacillus* strains with the use of antibiotics which represent the gold standard currently in use (Table 3). In the studies carried out by Sinol Sen et al. (2011; *B. subtilis* LS 1-2) and Froebel et al. (2020; DFM A: composed of *B. licheniformis* 21, *B. licheniformis* 842, and *B. subtilis* 2084; DFM B: composed of *B. subtilis* 747 and *B. subtilis* 1781), the use of Bacillus as a probiotic improved growth performance as compared with the basal control diet and equally with the use of antibiotics, such as avilamycin and bacitracin methylene disalicylate (Sinol Sen et al., 2011: −5% of the feed conversion rate [FCR] as compared with the control with the basal diet; Froebel et al., 2020: −2.4% of the FCR as compared with the control group with the basal diet). Furthermore, in 3 out of 8 studies, the probiotic group performed better than the antibiotic group. The data reported by Lee et al. (2014) showed that the BW of broilers supplemented with *B subtilis* was higher than the BW of broilers treated with salinomycin (Cumulative body weights at day 28 of *B. subtilis*-fed chickens 1276 ± 35.6 g vs. salinomycin 1172 ± 28.1 g). Similarly, BW and BWG were also significantly improved in broilers fed a probiotic mixture containing *B. subtilis* PB6 as compared with broilers treated with maduramicin ammonium (Teo and Tan, 2006; ADG was + 7.5% from day 21 to day 41 as compared with the antibiotic). *B. licheniformis* DSM 28710 also improved BWG (725 vs. 705 g from day 11 to day 22) and the FCR of broilers (1.41 vs. 1.42 from day 11 to day 22 and 1.50 vs. 1.52 from day 1 to day 36) as compared with salnomycin (Trela et al., 2020). However, it is not possible to establish which species/strain of *Bacillus* spp. might be the most promising to replace antibiotics, at least in prophylactic use. The evidence reported regarding the positive effect of some *Bacillus* strains for replacing antibiotics opens promising perspectives regarding the use of probiotics as an effective prophylactic strategy as well as growth promoters in broilers.

**TABLE 3:** The effect of Bacillus probiotics on the growth performance parameters of broilers as compared with antibiotic use.

N. animals	Challenge	Antibiotic	Probiotic	BWG^1^	BW^2^	ADG^3^	FI^4^	ADFI^4^	F:G (FCR)^5^	References
2280	No	Bacitracin methylene disalicylate	DFM A: *B. licheniformis* 21, *B. licheniformis* 842, and *B. subtilis* 2084; DFM B: *B. subtilis* 747 and *B. subtilis* 1781		> (DFM A)	> CO and = AB		=	>	Froebel et al., 2020
280	No	150 g per ton feed Avilamycin	Probiotic mixture^6^			=		=	=	Kazemi et al., 2019
135	No	60 mg/kg of salinomycin.	*B. subtilis*		> AB					Lee et al., 2014
320	No	20 mg/kg Avilamycin.	*B. subtilis* LS 1-2	>		>	>		>	Sinol Sen et al., 2011
600	*E. coli*	16.7 mg/kg bacitracin zinc + 3.3 mg/kg colistin sulfate.	*B. subtilis* PB6	>		> (infected and uninfected)			> (infected and uninfected)	Teo and Tan, 2006
400	No	Salinomycin addition (60 mg/kg diet	*B. licheniformis* DSM 28710	>		>	=		<	Trela et al., 2020
460	Eimeria maxima oocysts *C. perfringens*	65 g/MT of Narasin	*B. subtilis* DSM 32315		=		=		<	Whelan et al., 2019
480	No	75 mg/kg chlortetracycline	*B. coagulans*		> then CO and = AB	> than CO and = AB	=		=	Zhang et al., 2021

*BWG^1^: Body weight gain; BW^2^: Body weight; ADG^3^: average daily gian; FI^4^: Feed intake; ADFI^4^: average daily feed intake; F:G (FCR)^5^: feed to gain ratio; Probiotic mixture^6^: E. faecium, L. delbruekii subp.bulgricus, L. acidophilus, L. plantarum, L. rhamnosus, Bifidobacterium bifidum, S. salivarius thermuphilus, Candida pintolopesii and Aspergillus oryzae.*

## Conclusion

The present review shows that *Bacillus* strains applied as potential probiotics have been extensively studied in monogastric livestock species. Looking at the literature, historically, attention was focused on several strains of *B. subtilis* and in recent years, other strains have gained attention. Moreover, the passage from a mono-strain approach to a multi specie/multi-strain approach to exploit the synergistic capacity among different strains to face the complex issue of replacing the antibiotics used both in the prophylaxis or as growth promoters is evident. Globally, the data reported have demonstrated a complex and positive interplay between *Bacillus* spp. and the host which positively influenced feed digestion, regulation of the gut microbial communities, physiology, and the immune systems of both swine and poultry. A sufficient number of studies regarding post-weaning piglets and broilers have evidenced promising results in reducing the use of antibiotics by supplementation with Bacillus probiotics. In particular, it has emerged that Bacillus probiotics can favor growth in terms of the ADG of both post-weaning piglets and broilers, and can reduce the incidence of post-weaning diarrhea of pigs by 30% and the mortality of broilers by 6–8% as compared with the negative control groups at the same level as antibiotics. Furthermore, especially in broilers, an increase in gut health in terms of an increase in villus height (increased by 4–19% in the ileum), and a decrease in oxidative stress could be ascribed to the use of *Bacillus* strains. There is less consensus regarding the effect of Bacillus probiotics on sow performance in terms of BW and feed intake while a reliable beneficial effect on the reproductive parameters, namely litter BW and ADG during suckling, has been evidenced.

It is difficult to make concrete suggestions regarding specific strains of *Bacillus* in order to provide a practical guide to the field as the number of studies selected for the present review for each probiotic strain was not sufficient to carry out a thorough analysis. Therefore, to assess whether or not specific strains can achieve the same productivity gains and health protection on par with antimicrobials for a reduction in the use of antimicrobials, future studies including a control group treated with an antimicrobial are encouraged.

In summary, the present review confirmed the efficacy of *Bacillus* strains as a probiotic and as a potential tool for reducing antibiotic use in young pigs and chickens. Future research and developments provided from research institutions and the industrial sector should aim at identifying new strains of probiotics and defining the specific properties of probiotics in order to favor their target use in animals, based on their specific requirements during the different growing phases as well as specific sanitary conditions/pathogen infections. Continuous research and development regarding probiotics will help support a decrease in the use of antibiotics in livestock production and promote a more sustainable rearing system in the near future as requested by the farm to fork European approach, especially for broilers and piglets which are responsible for the higher in-feed antibiotic use in the livestock production system.

## Author Contributions

DL: original draft preparation, figure and table conceptualization, review and editing. PB and LR: review. LA: original draft preparation, table preparation and editing. SV: table preparation and editing. PT: original draft preparation and review. All authors contributed to critically revising the manuscript and gave final approval for publication.

## Conflict of Interest

The authors declare that this study received funding from Chr. Hansen, and Animal Health and Nutrition. The funder was not involved in the study design, collection, analysis, interpretation of data, the writing of this article or the decision to submit it for publication.

## Publisher’s Note

All claims expressed in this article are solely those of the authors and do not necessarily represent those of their affiliated organizations, or those of the publisher, the editors and the reviewers. Any product that may be evaluated in this article, or claim that may be made by its manufacturer, is not guaranteed or endorsed by the publisher.

## Footnotes

1
https://lpsn.dsmz.de/search?word=bacillus
